# A value-based deep reinforcement learning model with human expertise in optimal treatment of sepsis

**DOI:** 10.1038/s41746-023-00755-5

**Published:** 2023-02-02

**Authors:** XiaoDan Wu, RuiChang Li, Zhen He, TianZhi Yu, ChangQing Cheng

**Affiliations:** 1 Smart Health Laboratory, Hebei University of Technology, Tianjin, China; 2 College of Management and Economics, Tianjin University, Tianjin, China; 3 Emergency Department, Tianjin Medical University General Hospital, Tianjin, China; 4 Department of Systems Science and Industrial Engineering, State University of New York, Binghamton, NY USA

**Keywords:** Biomedical engineering, Computational models, Machine learning, Infection

## Abstract

Deep Reinforcement Learning (DRL) has been increasingly attempted in assisting clinicians for real-time treatment of sepsis. While a value function quantifies the performance of policies in such decision-making processes, most value-based DRL algorithms cannot evaluate the target value function precisely and are not as safe as clinical experts. In this study, we propose a Weighted Dueling Double Deep Q-Network with embedded human Expertise (WD3QNE). A target Q value function with adaptive dynamic weight is designed to improve the estimate accuracy and human expertise in decision-making is leveraged. In addition, the random forest algorithm is employed for feature selection to improve model interpretability. We test our algorithm against state-of-the-art value function methods in terms of expected return, survival rate, action distribution and external validation. The results demonstrate that WD3QNE obtains the highest survival rate of 97.81% in MIMIC-III dataset. Our proposed method is capable of providing reliable treatment decisions with embedded clinician expertise.

## Introduction

Sepsis is a life-threatening syndrome triggered by a chain reaction through the body to an infection. Absent timely intervention and treatment could lead to tissue damage, metabolic dysfunction, and acute organ failures^1^. Almost any infection, including COVID-19, can lead to sepsis. Approximately 30% of patients diagnosed with severe sepsis do not survive^2^. According to the international sepsis guidelines, fluid resuscitation and vasopressors are oftentimes administered to contain the infection, the dose of which should be adjusted according to dynamic measurements of the disease progression^3,4^. However, it remains a confounding quest in clinical practice to decide the optimal solution and dose of fluid and vasopressor therapy, particularly considering the individual difference. There is a lack of tools for personalized real-time decision support on sepsis treatment. The recent advances in electronic medical records (EMR)^5^ have provided an unprecedented opportunity to capture the evolution of patient health status and design cost-effective treatment plans^6^. Consequently, data-driven and artificial intelligence (AI) approaches, including supervised learning (SL)^7–9^ and reinforcement learning (RL)^10–12^, have been extensively attempted to assist clinical decision making^13^.

The dynamic treatment decision for sepsis is naturally a problem of Markov Decision Process (MDP)^14^. Komorowski et al. developed an RL approach based on the SARSA (State-Action-Reward-State-Action) algorithm^15^ to provide personalized treatment decisions for adult sepsis patients in intensive care unit (ICU)^16^. Here, the action is referred to as the dose of intravenous fluids and vasopressors, and the dynamic patient health status is considered as the state, which can be inferred from the physiological data. Yet, this method is only limited to a discrete state space, not amenable to the continuously evolving physiological status^17^. To address this limitation and avoid the curse of dimensionality in Q learning, approximation of the Q value has been extensively investigated in value-based deep reinforcement learning (DRL) algorithms, such as Deep Q-Network (DQN)^18^, Double Deep Q-Network (DDQN)^19^, Dueling Deep Q-Network (Dueling DQN)^20^ and Dueling Double Deep Q-Network (D3QN)^17^. Q value function elucidates the value to perform a given action in a given state. Such a value for the next state after taking an action is denoted as the target Q value, and an accurate estimation of the target Q value is crucial to policy improvement. However, if the estimation of the target Q value is inaccurate, overestimation or underestimation is likely to occur. For instance, the Dueling DQN structure follows the maximum target values and uses the same parameters in main network and target network to select and evaluate an action, which tends to the overestimation issue^21^. The D3QN structure possesses two neural networks with two separate sets of weights: the main network selects the optimal action, and then the target network computes the corresponding Q value for the action. We note that D3QN structure often selects sub-optimal actions for the target network, which tends to underestimate the target Q value^22,23^.

In spite of the recent leap forward in AI-boosted smart healthcare, it remains a challenging task for AI to outperform experienced clinicians in the diagnosis and treatment for a variety of diseases, including sepsis. Indeed, AI-derived systems cannot replace the physician in the clinical management of sepsis. The blind trust of AI algorithms in decision making for healthcare management without clinician supervision has led to increased medical risks and safety issues^24,25^. Remarkably, AI or data-driven models suffer from biases in data and model building, and consequently may provoke treatment solutions that are against the principle of clinic practices. To this end, hybrid systems of SL and RL that capitalize on the availability of large-scale EMR have been proposed, which are capable of providing reliable medical recommendations^26^. Nevertheless, the usage of SL not only increases the computational complexity but also limits the self-adaptiveness of the RL decision in long-term reward^27^. In addition, most existing studies on sepsis treatment hinge on a large number of features extracted from EMR, including blood glucose and white blood cell count, which could further impinge on the performance and interpretability of AI models. Thus, eliminating redundancy and singling out the most representative features are vital for RL agent to make precise perceptions. Therefore, we aim to integrate a DRL model with human expertise and sort out a critical subset of the clinical features in sepsis towards reliable and more clinically interpretable decision making for sepsis treatment.

**Fig. 1:**
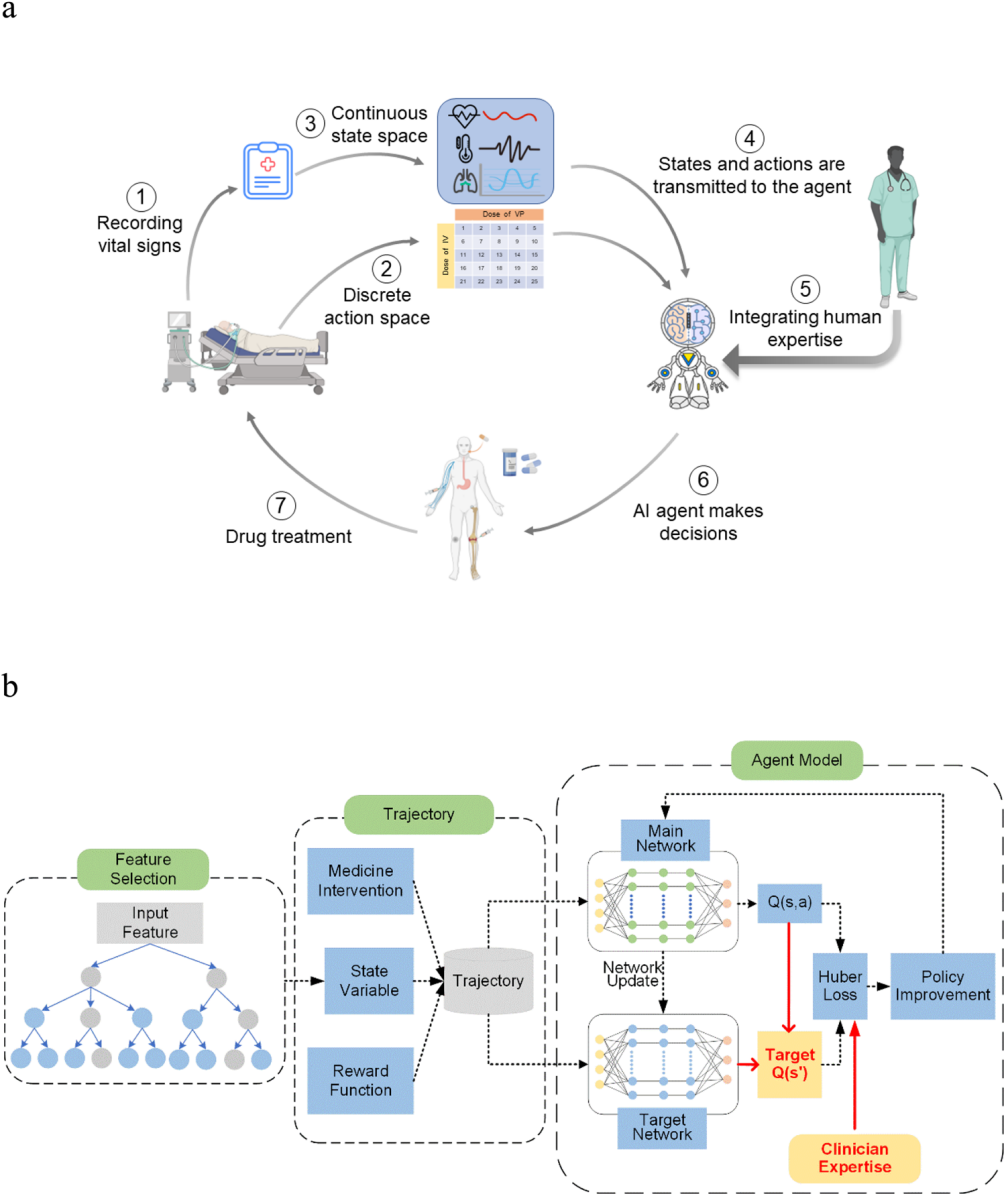
Architecture of WD3QNE algorithm. **a** The dynamic treatment process of the WD3QNE agent for sepsis. The continuous state space and discrete action space are then constructed. The DRL agent takes actions based on the current state and clinician expertise. **b** WD3QNE algorithm structure.

More specifically, we propose a Weighted Dueling Double Deep Q-Network with embedded human Expertise (WD3QNE) to aid real-time sepsis treatment. The algorithm architecture is shown in Fig. 1. Structure shows feature selection, trajectory, and agent model training. The innovations boil down as follows: (1) We develop a novel target Q value function with adaptive dynamic weight, which improves the accuracy of target Q value estimation and results in a higher-precision reinforcement learning model. The method makes a trade-off between Dueling DQN overestimation and D3QN underestimation in value estimation. It is worth noting that this method can be easily generalized to other value-based DRL methods. (2) An AI platform is constructed that integrates human expertise with the DRL model: the human expertise provides guidance for AI and ensures higher efficiency and reliability in sepsis treatment. We offer novel insights for incorporating human expertise with DRL. (3) The important features of clinical relevance for septic patients are selected by a random forest algorithm. We eliminate statistical redundancy among those commonly used clinical and biological features and enhance the clinical interpretability of DRL. We also compare WD3QNE with other widely used value-based DRL methods, including DQN, DDQN, D3QN, and WD3QN, in terms of the expected return, survival rate and action distribution treatment for sepsis using the MIMIC-III dataset^28^. We demonstrate that the WD3QNE policy outperforms human clinicians and other value-based DRL methods and achieves the highest survival rate. We further compare the drug intervention distribution of a pure AI and AI with embedded human expertise. In addition, to explore the generality of the target Q value function with adaptive dynamic weight as proposed in this paper, we use OpenAI Gym LunarLander-v2 environments^29^ to validate our model’s performance (see Supplementary Note 1).

## Results

### Q value function

In this study, we utilize a dueling deep neural network framework for both the main network and the target network to approximate the Q function, $Q\left( {S_t,a_t} \right)$. The dueling network structure has two streams to separate out the estimate of state value *V* and the advantage *A* for each action^30^ as $Q\left( {S_t,a_t} \right) = V\left( {S_t} \right) + A\left( {S_t,a_t} \right)$. The dueling architecture learns the states that are valuable or not, without having to learn the value of each action for each state. Here, the state *S*_*t*_ represents the health status of the patients and the action *a*_*t*_ is the prescribed dose of intravenous fluids and vasopressors at time *t*. The agent takes an action *a*_*t*_ in current state *S*_*t*_ and transitions to the next state *S*_*t*+1_. In Dueling DQN, the target Q values are derived from the target network under the state $S_{t + 1}$ at time *t* + 1. The maximum Q value is then selected, which tends to result in overestimation^30^. In D3QN, the action is first determined by the main network, and then the target Q value is obtained from the target network, which tends to result in underestimation^31^. Hence, the target Q value estimation in both Dueling DQN and D3QN can be inaccurate, largely owing to the uncertainty of the action in the next state. To find more accurate target Q value estimation, we design a novel target Q value function with adaptive dynamic weight *p* (Eq. (1)) to realize a trade-off between Dueling DQN and D3QN to derive the optimal policy:

1 $$Q\left( {S_{t + 1},a_{t + 1}} \right) = p \times \mathop {{\max }}\limits_{a_{t + 1}} Q\left( {S_{t + 1},a_{t + 1};{{{\mathrm{\omega }}}}^ - } \right) + \left( {1 - p} \right) \times Q( {S_{t + 1},\mathop {{{{{\mathrm{argmax}}}}}}\limits_{a_{t + 1}} Q\left( {S_{t + 1},a_{t + 1};\omega } \right);\omega ^ - } )$$

where *ω* are the parameters of the main network, and *ω*^−^ are the parameters of the target network. The adaptive dynamic weight *p* (Eq. (2)) is calculated as:

2 $$p = \frac{{\varphi _{a_{t + 1}}}}{{\varphi _{a_{t + 1}} + \sigma _{a_{t + 1}}}}$$

Here, $\varphi _{a_{t + 1}}$ is the maximum target Q value divided by the summation of the target Q value under all possible actions (Eq. (3)), and the target Q values are obtained from the Dueling DQN method. Similarly, the dynamic parameter $\sigma _{a_{t + 1}}$ is obtained from the D3QN method (Eq. (4)).

3 $$\varphi _{a_{t + 1}} = \frac{{\mathop {{\max }}\limits_{a_{t + 1}} Q\left( {S_{t + 1},a_{t + 1};\omega ^ - } \right)}}{{\mathop {\sum}\nolimits_{a_{t + 1}} {{{\mathrm{Q}}}} \left( {S_{t + 1},a_{t + 1};\omega ^ - } \right)}}$$

4 $$\sigma _{a_{t + 1}} = \frac{{Q(S_{t + 1},\mathop {{{{{\mathrm{arg}}}}\max }}\limits_{a_{t + 1}} {\it{Q}}\left( {S_{t + 1},a_{t + 1};\omega } \right);\omega ^ - )}}{{\mathop {\sum}\nolimits_{a_{t + 1}} {Q(S_{t + 1},} \mathop {{{{{\mathrm{arg}}}}\max }}\limits_{a_{t + 1}} {\it{Q}}\left( {S_{t + 1},a_{t + 1};\omega } \right);\omega ^ - )}}$$

We use the adaptive dynamic weight to seek the balance of the estimated target Q values of the two methods, so that the approximate value of target Q is closer to the unbiased estimator. The Q value function $Q\left( {S_t,a_t} \right)$ is the expected cumulative reward from taking a certain action *a*_*t*_ in state *S*_*t*_ following a policy. In order to estimate the Q value of current state *S*_*t*_, we add the reward for performing an action *a*_*t*_ to the target Q value $Q\left( {S_{t + 1},a_{t + 1}} \right)$. Finally, the Q value function (Eq. (5)) is obtained.

5 $$Q\left( {S_t,a_t} \right) = r + \gamma Q\left( {S_{t + 1},a_{t + 1}} \right)$$

where *r* is the reward after performing an action in the state *S*_*t*_ (see Reward function), and *γ* is the discount factor.

Additionally, the personalized treatment of sepsis is a complex puzzle for clinical management^14^. It is crucial to ensure the reliability and safety of therapeutic interventions under personalized treatment planning. Nonetheless, a DRL agent only interacts with the environment to seek the optimal actions with high reward, regardless of the potential risks. It has been noted that certain actions induced by AI could cause high risk and lead to contentious medical solutions^26^, which has significantly stymied the broad adoption of AI in healthcare management. On the other front, human experts maintain an edge over AI in abstract reasoning under ambiguous conditions. Thus, a trend of keeping human in the loop in critical decision making has been emphasized in a host of industries domains. Here, we guide the DRL agent to perform actions by incorporating human expertise. Raghu et al. found that for sepsis patients with mild symptoms, the more similar a pure AI policy is to a clinician’s policy, the greater the patient’s survival rate. Thus, human clinicians are more reliable than pure RL agents in this scenario, which is partially owing to the fact that human clinicians are more cautious about other issues including individualized health status and drug interactions. Interestingly, such disparity does not exist for patients with severe symptoms. For patients with severe symptoms, the optimal treatment strategy is still in the infancy stage^31^, and not too much human expertise can be utilized for comparison or to guide the AI. Komorowski et al. analyzed the drug dose distribution, and found that AI policy tended to give high doses of the vasopressor^32^. Particularly, in the latest guideline on sepsis management, an initial target value of 65 mm Hg for the mean-arterial-pressure (MAP) has been suggested in lieu of 72.6 mm Hg as previously recommended^3^. That said, a high dose of vasopressors is no longer favored in the initial stage. Furthermore, Raghu et al. divided the Sequential Organ Failure Assessment (SOFA) scores into three levels (<5, 5–15, and >15) to evaluate model performance for different severity subcohorts^17^. Here, we employ the human clinician expertise at the lowest SOFA level and along with the patient outcome to estimate the target Q value function and guide the agent. We propose the Q value function of clinician expertise (Eq. (6)):

6 $$Q^{clin}\left( {S_t,a_t^{clin}} \right) = r + \gamma Q^{clin}\left( {S_{t + 1},a_{t + 1}^{clin};\omega ^ - } \right)$$

Accordingly, if SOFA is < 5, we use the Q value function of clinician expertise, otherwise the novel Q value function is leveraged. The Q value function of WD3QNE algorithm is given by:

7 $$Q^{WD3QNE} = \left\{ {\begin{array}{*{20}{c}} {Q^{clin}\left( {S_t,a_t^{clin}} \right)} \\ {Q\left( {S_t,a_t} \right)} \end{array}} \right.\begin{array}{*{20}{c}} {if\;SOFA < 5} \\ {otherwise} \end{array}$$

### Survival rate and safety rate

We first calculate the expected return based on the double robust off-policy value evaluation using the MIMIC-III dataset^28^. We choose several value-based DRL algorithms for comparison with our WD3QNE: DQN^22^ combines Q learning with a deep neural network; DDQN^23^ is a variant of deep Q learning with two neural networks, main network and target network; D3QN^31^ is DDQN combined with Dueling DQN; Weighted Dueling Double Deep Q-Network (WD3QN) introduces a target Q value function with adaptive dynamic weight into D3QN, but does not use the human expertise. Compared to other value-based DRL, the target Q value function is additionally adopted to revise the Q value function in WD3QN. We divide the MIMIC-III dataset into training set (80%), validation set (10%) and test set (10%). Experimental studies are conducted and the algorithms are run 30 times.

We obtain the survival rate according to the return value (see Methods). The expected return and the survival rate on the test dataset are shown in Table 1. The results show that the AI policy has a higher survival rate than the human clinician’s policy. The feature selection process improves the performance of algorithms, because all models with feature selection (37 features) achieve better performance than the same type of models without feature selection (45 features). With 37 features, it is noteworthy that the WD3QNE obtains the highest survival rate of 97.81% with the lowest standard deviation of 0.0012. The results showed that the survival rate of the human clinician’s policy is 83.26% with expected return 14.11. The survival rate of D3QN policy is 96.48% with expected return 22.27. The survival rate of WD3QN policy is 97.49% with expected return 23.08. Overall, to human clinicians, WD3QNE survival rate is improved survival by 17.5%. The WD3QNE survival rate is also an improvement of 1.38% compared to D3QN and 0.32% compared to WD3QN.

**Table 1:** Off-policy evaluation performance of baselines in the test set.

Methods	Expected return	Survival rate (%)
Clinician	14.11	83.26
DQN-45	20.37 ± 0.32	88.29 ± 0.15
DDQN-45	20.17 ± 0.45	88.33 ± 0.26
D3QN-45	21.52 ± 0.34	89.35 ± 0.41
DQN-37	20.92 ± 0.21	94.29 ± 0.46
DDQN-37	20.20 ± 0.44	93.59 ± 0.19
D3QN-37	22.27 ± 0.30	96.48 ± 0.56
WD3QN-37	23.08 ± 0.19	97.49 ± 0.14
WD3QNE-37	23.63 ± 0.15	97.81 ± 0.12

**Fig. 2:**
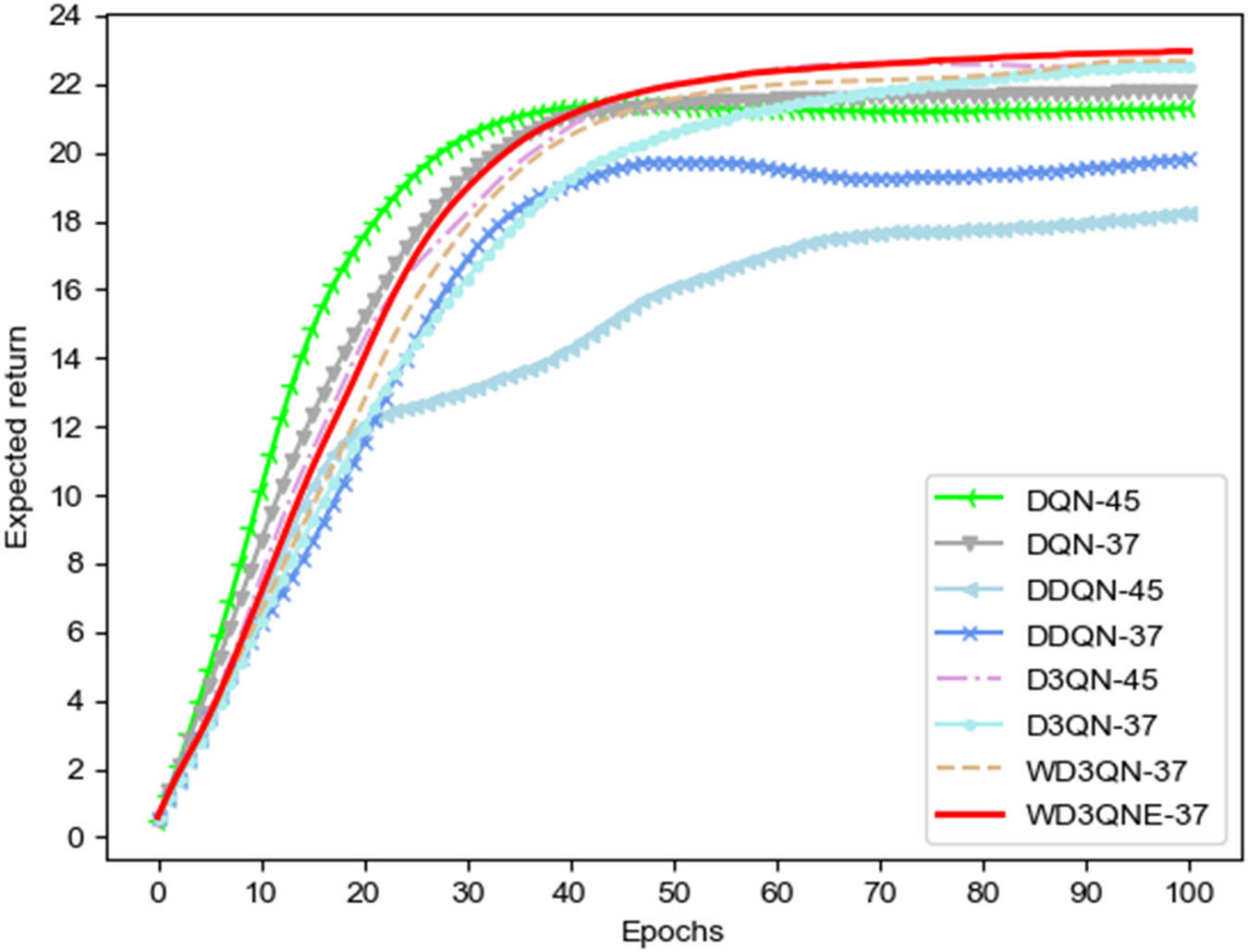
Expected return of different algorithms at each learning epoch. The value-based DRL algorithms is run for 100 epochs in the validation set with feature selection (37 observation features) and without feature selection (45 observation features). Number 37 means 37 observation features that we select with the random forest algorithm. Number 45 means 45 observation features. Although the DQN algorithm converges fast in the beginning, it exhibits premature convergence.

Furthermore, in Fig. 2, we present the expected return of different algorithms at each learning epoch in the validation set. The WD3QNE expected return values converge and stabilize around reward value 24. Our proposed method outperforms other baseline methods. It is noteworthy that WD3QNE with human expertise achieved better performance than WD3QN without human expertise. Additionally, although the DQN algorithm has the fastest convergence in early period, it converges to the local optimal value.

### Action distribution

**Fig. 3:**
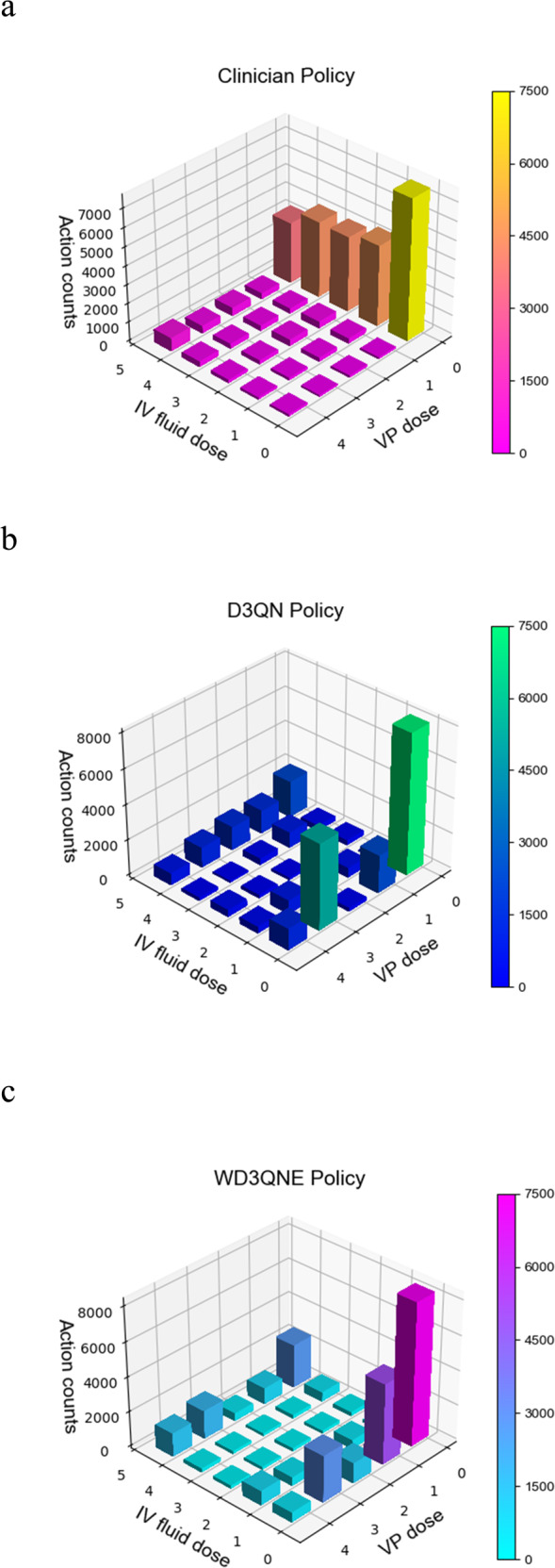
Action distribution for the test set. **a** Action distribution of the human clinician policy. **b** Action distribution of the DQ3N policy with 37 observation features. **c** Action distribution of the WD3QNE policy with 37 observation features. We aggregate all actions selected over all timesteps for the five dose bins of both medications. 0 denotes no drug given. We discretize the action space into per-drug quartiles. Action counts represent the utilized times of the drug dose. We can see that the human clinician policies tend to use low doses of vasopressors. The pure AI clinician policies (D3QN) tend to use high doses of vasopressors. The AI clinician policies with embedded human expertise (WD3QNE) tend to use lower doses of vasopressors than D3QN and higher doses of vasopressors than the clinician.

For further interpretation, we demonstrate the optimal policies derived from the three representative methods (human clinician, D3QN and WD3QNE). The action distribution of the clinician policy is given in the MIMIC-III set. As shown in Fig. 3, the clinician uses low doses of vasopressors, while D3QN uses higher doses of vasopressors than the clinician. Obviously, the AI policy is very different from that of the clinician. If the sepsis is mild, when we introduce human expertise to the AI agent, we find that the WD3QNE policy uses lower doses of vasopressor than the pure AI policy. Although vasopressors are commonly used in the ICU to increase MAP, most sepsis patients do not need high doses of vasopressors^3^. The WD3QNE model provides personalized treatment decisions based on the patient’s dynamic response.

### Sensitivity analysis

**Fig. 4:**
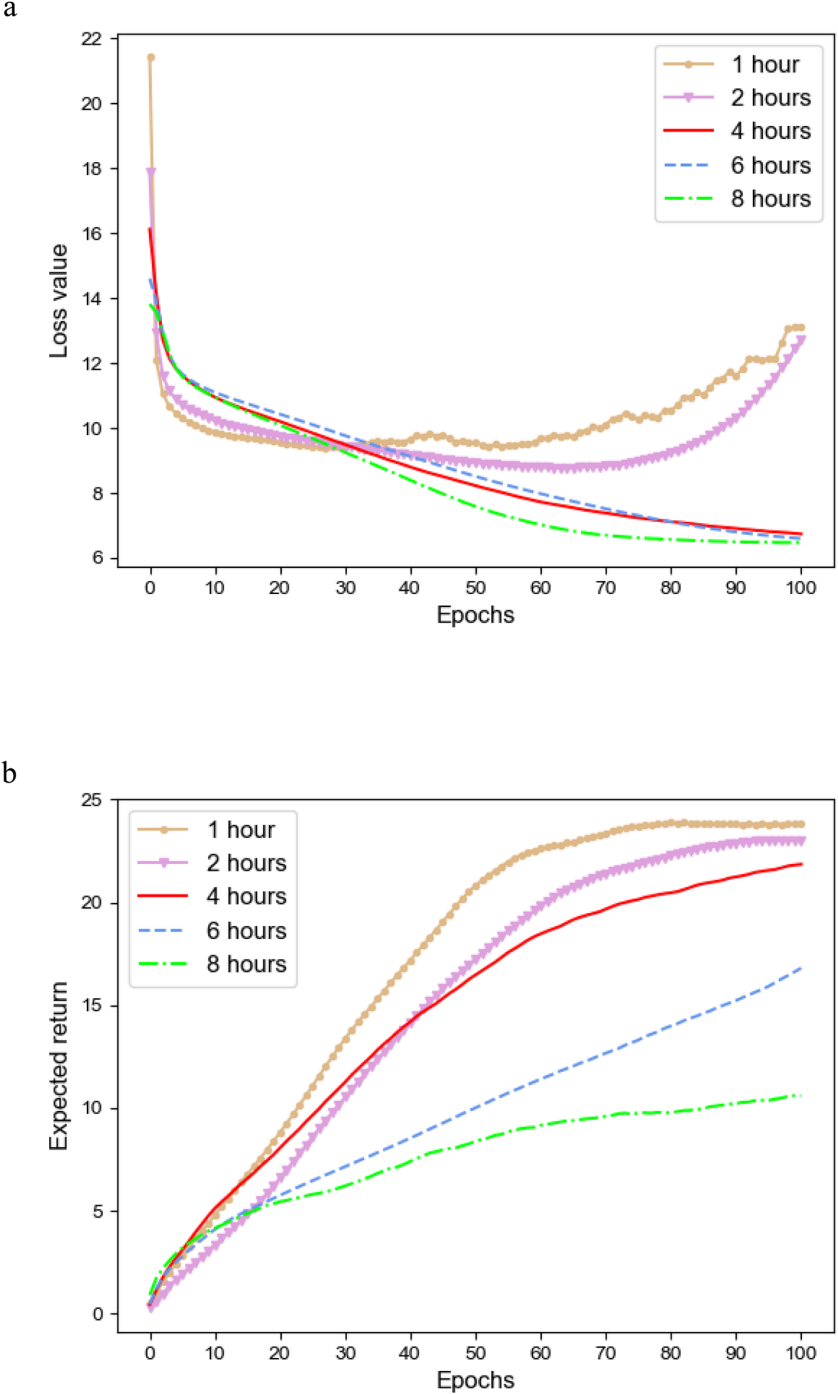
Performance results of different binning intervals at each learning epoch. The patient trajectories are discretized into different binning intervals, 1 h, 2 h, 4 h, 6 h, and 8 h. **a** The loss value of different binning intervals in training. **b** The expected return of different binning intervals in the training test.

To analyze the effect of patient information coded as discrete time series into different hours, we perform a sensitivity analysis for the binning intervals of 1 h, 2 h, 4 h, 6 h, and 8 h. As shown in Table 2, the maximum records 1,104,929 and minimum records 138,116 in the training set are used, and therefore we set the batch size of patients to 32 and 256 in training respectively. Specially, to ensure the fairness of the test set, we included 100 patients in the test data and each patient has 5 samples for different binning intervals. The test set has 9768 records. The performance results of different binning intervals at each learning epoch are shown in Fig. 4. We can see that the smaller intervals obtain larger expected value and faster convergence in test set, which captures finer state changes. The binning intervals of 1 h and 2 h has a lot of missing values and therefore can result in overtraining. In total, the more frequent state data make the model better in the case of fewer missing values.

**Table 2:** Records of different binning intervals.

Hour bins	Batch size	Records of training set	Records of test set
1 h	32	1,104,929	9768
2 h	64	552,464	9768
4 h	128	276,232	9768
6 h	192	184,153	9768
8 h	256	138,116	9768

### External validation

We conduct an external validation using the eICU Research Institute Database (eRI) from Philips^33^. A total of 1500 sepsis patients with fewer missing values are select. Using the methods suggested by Komorowski^32^, hospital mortality is considered as the final outcome in this cohort. We extract the 24,279 records, which spans the time interval of 36 h preceding and 72 h after the estimated onset of sepsis. The off-policy evaluation performance in the eRI dataset is shown in Table 3. The results show that the survival rate of WD3QNE policy is 95.83% with expected return 21.81. The WD3QNE with human expertise achieve better performance than other algorithms.

**Table 3:** Off-policy evaluation performance of baselines in the eRI set.

Methods	Expected return	Survival rate (%)
DQN-37	19.82	93.44
DDQN-37	19.94	93.57
D3QN-37	20.74	94.27
WD3QN-37	21.13	94.62
WD3QNE-37	21.81	95.83

### Bellman error tracking

**Fig. 5:**
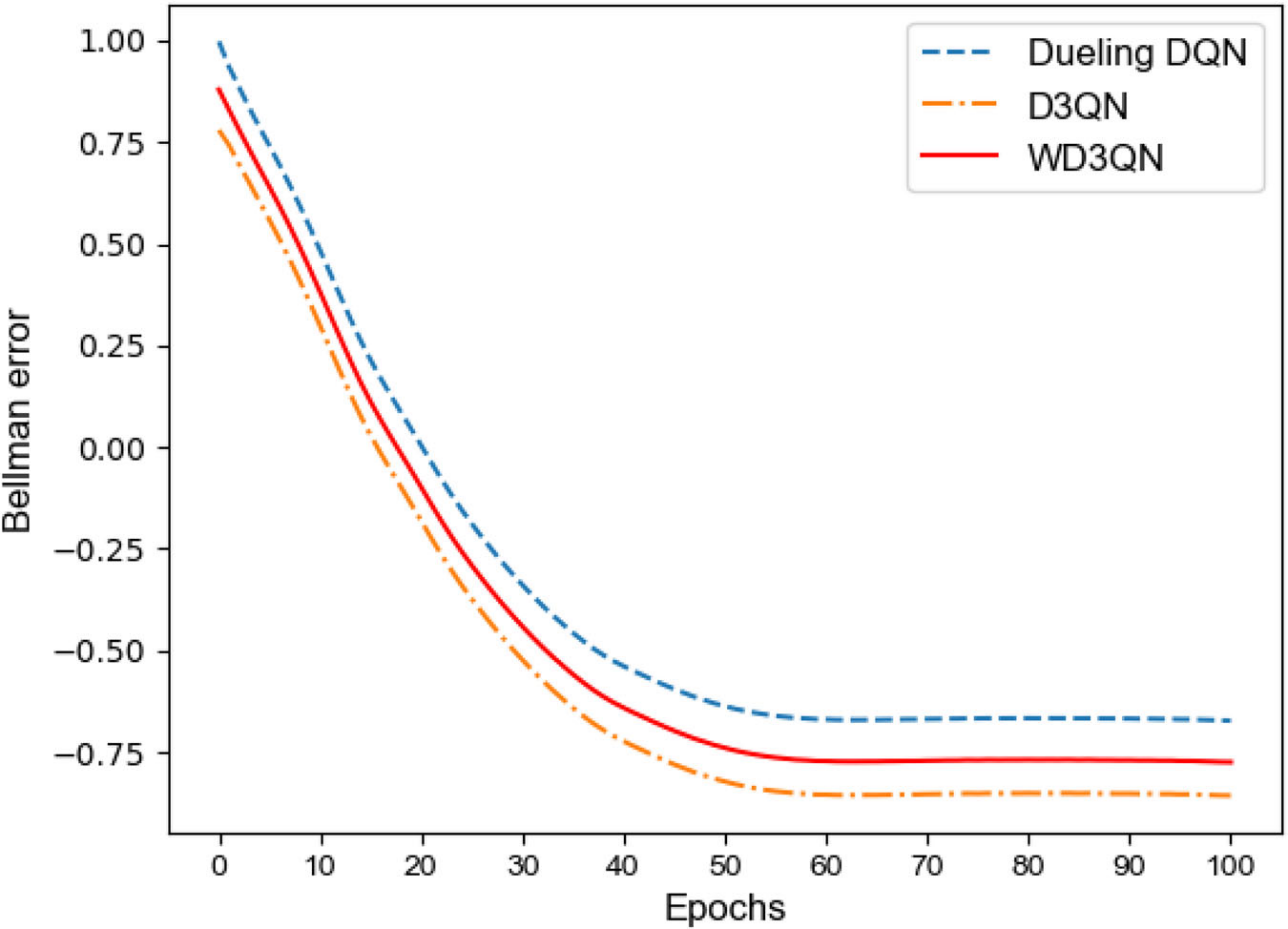
Bellman error as a function of epochs. Visualization of Bellman error evolution. The WD3QN is shown in red. The Dueling DQN is shown in blue while the D3QN is shown in orange.

For insightful analysis about how the algorithm behaves, we track the Bellman error through training in Fig. 5. The result shows that the Bellman error will gradually decrease with the iteration and finally stabilize. Similarly, we find that *p* varies between 0 and 1. At last, *p* is close to 0.5. The target Q value of WD3QN value is between the target Q value of Dueling DQN and the target Q value of D3QN.

## Discussion

We proposed the WD3QNE algorithm with a novel value function and integration of clinician expertise of human clinicians for septic patients in the ICU. As shown in Table 1 and Fig. 2, the WD3QNE algorithm outperforms the conventional DRL approaches. Compared to the human clinician and pure AI algorithm, our model learns an optimal policy and notches reliable treatment action distribution in Fig. 3.

To address the bias issue in Q value estimation, we design a target Q value function with adaptive dynamic weight (WD3QN) in the WD3QNE algorithm. In Table 1, we demonstrate that WD3QN method achieves a higher survival rate (97.49%) compared to D3QN (96.48%), along with significantly lower variability. This is attributed to the fact our target Q value function finds a trade-off between the Dueling DQN overestimation and the D3QN underestimation. Compared with other DRL methods^22,23,28^, this Q value function estimates the Q value of the next state more precisely without incurring additional algorithm complexity in this study. We further note that the WD3QN framework can be easily adopted to other problems. As demonstrated in Supplementary Note 1, it registers high performance with respect to optimization results and operation time.

In sepsis treatment, AI can be particularly beneficial in assisting decision-making processes. However, DRL agents tend to seek the maximal rewards via aggressive strategies, incurring extra risks for patients in clinical practice. Moreover, it is still controversial, ethically and practically on how much we can follow the guidelines from AI, particularly when the AI solution deviates substantially from the human clinician’s policy. As aforementioned, it has been recognized that AI policy prescribes overdose of vasopressors in sepsis treratment^17^. In such cases, some may argue that it is necessary to bring in the expertise of human clinicians to help AI make comprehensive judgments for risk control. Human expertise tends to avoid so-called “common-sense mistakes” such as overdosing as well as be more cautious about other issues including side-effects and drug interactions, which are vital towards elevating survival rate. However, such abstract knowledge or expertise is largely missing in existing AI approaches. In contrast to SL deep neural network models^27,34^, the focus of our study is to set human expertise as constraints on feasible solutions for AI algorithms. We put to test that with such constraints, the proposed WD3QNE model prescribes reasonable doses of vasopressors (lower than those from alternative models without human expertise) for mild sepsis, as shown in Fig. 3. The algorithm with human expertise converges faster than other approaches without such human expertise, owing to the reduced search space for the optimization problem. Overall, the DRL algorithm with clinician expertise has achieved excellent performance by integrating the advantages of both AI and human clinicians, thus optimizing the allocation of medical resources as well as advancing AI technology in medical applications.

In addition, the high-dimensional ICU data on sepsis and septic shock are challenging for DRL to address. Komorowski et al. used a random forest^35^ classification model to rank the importance of the features. Their studies suggested the presence of some redundant features, so eliminating those features is also an important step in further training our algorithm for learning the patterns of disease. Therefore, we used a random forest as a feature selection method to narrow our feature set from the original 45 features, as suggested by Raghu et al.^31^. In total 37 features were selected as the final subset to achieve the best performance as shown in Table 1 and Fig. 2.

One limitation of this study is that our reward function only considered survival rate and SOFA score. The intermediate rewards and final rewards are essential components of RL algorithm. We would like our reward function to capture changes in and clinical significance of organ function of patients accurately. Therefore, in future studies, we will further collect treatment data on sepsis and expert advice to design a better reward structure. Additionally, collecting large amounts of data from the real world or simulator, the agent can greatly reduce sample efficiency and lead to unexpected behavior. We use historical data to learn the rules and teach RL agent to complete the tasks. Usually, the historical data are time series trajectory of human behavior. RL agent learns the optimal policy at a state from different clinicians and extracts implicit knowledge from a large offline data set. The agent can cause extrapolation errors from out-of-distribution actions and is not exploring. A lot of offline data of human behavior or a suitable regularization item of offline RL are needed. We will investigate the offline RL algorithmic in sepsis treatment problem in our future work. In the actual treatment process, doctors should formulate treatment schedules according to the physical or emotional needs of patients. AI-integrated treatment methods should thus also consider personalized needs of the doctor or the patient, such as minimal cost, minimal side effects and minimum ICU stay. Moreover, as we have demonstrated in this study, the inclusion of human expertise in the case of SOFA < 5 improves the survival rate. A full-scale integration of human expertise (e.g., drug interaction, side effect and common sense) in the decision making loop will be further investigated.

## Methods

### Dataset

We use the dataset from the Multiparameter Intelligent Monitoring in Intensive Care (MIMIC)-III v1.4 database^28^, which is a de-identified database of 61,532 admissions to the intensive care unit from 2001 to 2012 from the Beth Israel Deaconess Medical Center in Boston, Massachusetts, USA.

We exclude patients whose treatment was withdrawn or had missing records over 24 h. We select 276,232 records from 17,083 adults with SOFA greater than or equal to 2 according to the latest Sepsis definite-Sepsis 3.0^36^. As in previous studies, we use 45 physiological feature variables, including demographics, vital signs and lab values shown in Tables 4 and 5. In training, patient data and interventions are recorded every 4 h. We use 80 h of patient records from up to 24 h preceding until 56 h following the estimated onset of sepsis. As a result, the period T = 20. In the external validation, the maximum period T = 80 from the cohort monitored every hour. The outcome is 90-day mortality^37^.

**Table 4:** Demographics of sepsis cohort.

Category	Feature	(Mean, SD)	Feature	(Mean, SD)	Feature	(Mean, SD)
Demographics	All patients	17,083	Survivors	13,855	Non-survivors	3,228
	Male (*N*, %)	9,604 (56.2%)	Race	[W, B, A, L,	Age	64.4 (17.1)
	Weight	83.17 (24.6)		O]		
Vital signs	SOFA	6.3 (3.4)	SIRS	1.62 (1.04)	GCS	12.58 (3.43)
	HR	87 (16.7)	SBP	119 (20.3)	MBP	78.2 (13.4)
	DBP	57.1 (13.3)	Shock Index	0.74 (0.19)	SpO_2_(%)	96.9 (2.65)
	Temperature (°C)	36.9 (2.02)				
Lab values	Potassium	4.07 (0.55)	Sodium	138 (4.91)	Chloride	104 (6.27)
	Glucose	5.7 (1.1)	BUN	4.7 (2.3)	Creatinine	0.78 (0.23)
	Magnesium	1.11 (0.14)	Calcium	8.3 (0.79)	PaCO_2_	41.8 (10.7)
	SGOT	38.2 (12.6)	TB	10 (2.99)	WBC	8.2 (2.2)
	Platelets	224 (118)	PTT	31 (6.44)	PT	16 (6.64)
	INR	1.5 (0.82)	PH	7.3 (0.07)	PaO_2_/FiO_2_	248 (107)
	PaO_2_	99 (23.5)	HCO_3_	24 (5.06)	AL	2.05 (1.68)
	ArterialBE	0.33 (5.0)	RR	20 (5.18)	FiO_2_	0.45 (0.18)
	SGPT	31 (21.5)	HGB	10.2 (1.73)		
Fluid balance	Total input	5783 (4802)	Total output	4071 (4306)	4 Hourly output	387 (369)
	CB	1690 (1333)				

Race: White, Black, Asian, Latino, Others; *SOFA* Sequential Organ Failure Assessment, *SIRS* Systemic Inflammatory Response Syndrome, *GCS* Glasgow Coma Scale, *HR* Heart Rate, *SBP* Systolic Blood Pressure, *MBP* Mean Blood Pressure, *DBP* Diastolic Blood Pressure, *BUN* Blood Urea Nitrogen, *SGOT* Serum Glutamic-Oxaloacetic Transaminase, *SGPT* Serum Glutamic Pyruvic Transaminase, *TB* Total Bilirubin, *WBC* White Blood Cells Count, *PTT* Partial Thromboplastin Time, *PT* Prothrombin Time, *INR* International Normalized Ratio, *PH* Arterial Potential of Hydrogen, *PaO*_*2*_*/FiO*_*2*_ PaO_2_/FiO_2_ Ratio, *PaO*_*2*_ Partial Pressure of O_2_, *HCO*_*3*_ Bicarbonate, *AL* Arterial Lactate, *ArterialBE* Arterial Base Excess, *RR* Respiratory Rate, *FiO*_*2*_ Fraction of Inspiration O_2_, *SGPT* Serum Glutamic Pyruvic Transaminase, *HGB* Hemoglobin, *CB* Cumulated Balance.

**Table 5:** WD3QNE Algorithm.

Input: Electronic records $X \leftarrow x_1,x_2, \ldots ,x_j$, Action *A*, *M,N,γ,β*_*s*_*,β*_*T*_*,T*	
Output: Neural network weight ω^*^	
1:	Initialize *m* = 1, *n* = 1 and random main network weights *ω*, target network weights *ω*^-^
2:	Compute state reward *r* according to formula (9)
3:	Construct treatment trajectories $D \leftarrow \{ state\;S_t,\;a{{{\mathrm{ction}}}}\;a_t,\;reward\;r\}$, $t = 1, \ldots ,T$
4:	while *m* < *M* do
5:	for *n* < *N* do
6:	Select trajectories $\left\{ {S_t,a_t,r,S_{t + 1},a_{t + 1}} \right\} \leftarrow D$
7:	Compute target Q value $Q^{WD3QNE},Q^{clin},Q(S_{t + 1})$ according to Eqs. (1)–(7)
8:	Compute Q value $Q(S_t,a_t)$
9:	Compute the total loss *L* for the batch *n* using Huber loss
10:	Perform a gradient descent step $\omega ^{n + 1} = \omega ^n - \delta \nabla _\omega L$
11:	if *n*% 30 = 0 then
12:	Update target Q net $\omega ^ - = \omega ^{n + 1}$
13:	end if
14:	end for
15:	Set *m* = *m*+1
16:	end while
17:	Return ω^* ^= *ω*^*m*^

### Feature selection

Because the ICU observation data have many redundant features, the algorithm is more likely to suffer the curse of dimensionality^38^. Feature selection is a vital dimension reduction method for high-dimensional data^39^. Here, we employ a random forest model to select the vital features^35^. The random forest is an ensemble classifier composed of multiple decision trees. We randomly sample data by bootstrapping with resampling. Multiple decision trees are constructed for each resampling by the random splitting technique. The final prediction results are obtained through voting. The random forest has a high tolerance for outlier and noise. More importantly, the variable importance of each characteristic variable can be given. We employ the Out-Of-Bag (OOB)^40^ to measure the importance *G*_*i*_ of features *X*_*i*_ (Eq. (8)):

8 $$G_i = \frac{1}{B}\mathop {\sum }\limits_{j = 1}^B \left| {D_j - D_{ji}} \right|$$

where B indicates the number of samples. $i = 1,2, \ldots ,N$ is the *i*^*th*^ feature. *D*_*j*_ indicates the number of correct classifications by OOB, and *D*_*ji*_ indicates the number of correct classifications of samples after perturbation.

**Fig. 6:**
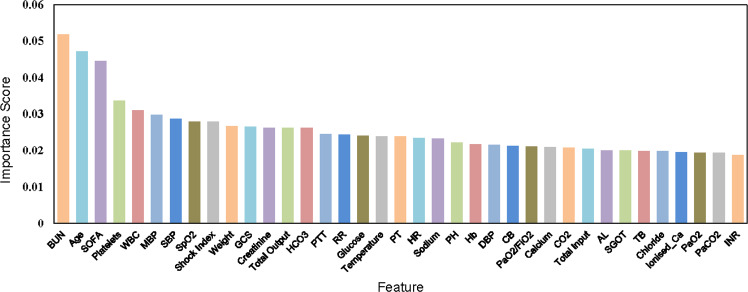
Feature importance score. We calculate the classification accuracy with death as the label for different numbers of features. The 37 features (variables) selected with the highest accuracy are displayed. The glossary of vital signs and lab values is provided in Table 4.

First, the importance scores are used for ranking all features. Second, the sequential backward search method is employed. The classification accuracy (Acc) is calculated with death as the label. Each time remove the least important feature with the lowest importance score from the feature set. Finally, we obtain 37 observation features with the highest classification accuracy, which will be used as the input of feature perception (see Fig. 6 for the ranking).

### State space and action space

Continuous state space is more sensitive to the subtle change embedded in the physiological data^41^. The state space is the 37 observed features consisting of vital signs and personal information after selecting features. We combine the observed states excluding the SOFA score into a state space as inputs to networks. The SOFA score is used as an intermediate reward in training. Additionally, we use a combination of intravenous (IV) fluid and vasopressor (VP) as the intervention action space for sepsis. The action takes place every four hours. We define a 5 × 5 action space^17^ for IV and VP. Except for zero doses of medicines as bin 0, we discretize the action space into per-drug quartiles and convert each drug at every timestep into an integer representing its quartile bin.

### Reward function

The reward function provides a flexible indicator to promote or punish specific actions of agents. The agent performs an action *a*_*t*_ in state *S*_*t*_ to reach the next state *S*_*t*+1_ and receives the reward *r*. We evaluate agents by associating reward with the target Q value function. Because only the patient’s survival is concerned, the reward is observed after a long sequence of decisions. We also apply intermediate rewards and final rewards in the form of SOFA change and survival after 90 days respectively^26^. SOFA represents the evidence of organ dysfunction and has been recommended by experts as a screening tool for sepsis^36^. Then we define the reward *r* as (Eq. (9)):

9 $$r = \left\{ {\begin{array}{*{20}{c}} {\beta _s \times \left( {SOFA_t - SOFA_{t + 1}} \right)} \\ {\delta\, \times\, \beta _T} \end{array}\begin{array}{*{20}{c}} {{{{\mathrm{t}}}} < {{{\mathrm{T}}}}} \\ {t = T} \end{array}} \right.$$

where *δ* means patient survival 1 or death -1. *β*_*T*_ is a final reward value 24 and *β*_*s*_ is a reward parameter.

### Dueling net architecture

Our paper adopts an off-policy reinforcement learning method based on value function. DQN, DDQN, and D3QN are the popular methods for off-policy learning^17–19^. The goal of those methods is to maximize the expected return. The value function and state-action value function are defined as $V^\pi (S_t) = {\Bbb E}[Q\left( {S_t,a_t} \right);\pi ]$ and $Q^\pi (S_t,a_t) = {\Bbb E}[r|S_t = S,a_t = a;\pi ]$. For instance DQN, the optimal Q function satisfies the Bellman equation: $Q^ \ast (S_t,a_t) = r + \gamma {\Bbb E}[\mathop {{\max }}\limits_{a_{t + 1}} Q\left( {S_{t + 1},a_{t + 1};\omega ^ - } \right)]$. In the sepsis environment, treatment effects rely on both the patient’s observed state and the doctor’s different intervention action. Thus, we use the dueling net architecture^30^ which maintains separate value and advantage functions: $Q^\pi \left( {S_t,a_t} \right) = V^\pi \left( {S_t,a_t} \right) + [A^\pi \left( {S_t,a_t} \right) - \frac{1}{{|A|}}\mathop {\sum }\nolimits_{a_t^\prime } A^\pi \left( {S_t,a_t^\prime } \right)]$. *V* is the value of the patient state and *A* is the advantage of prescription according to the specific policy π.

### Deep neural network

The neural network includes the input layer, the hidden layers with 256-dimensional fully connected layer, the hidden layers with 128-dimensional fully connected layer, the streams layer, and the output layer. All hidden layers are activated by rectified linear units (ReLUs). Dueling neural network parameters are updated by gradient descent according to Q value function shown in Eq. (7). In our paper, we use the Huber loss function^21^. This loss function combines the mean squared error function and the absolute value function. It divides the error into three segments. Between -1 and 1 use the mean square error (MSE), otherwise use absolute value. Putting all the aforementioned components together, the WD3QNE algorithm is provided in pseudocode WD3QNE Algorithm.

### Off-policy evaluation

In experiments, we use the intermediate reward parameter *β*_*s*_ = 0.6 and the terminal reward parameter *β*_*T*_ = 24, following the setting in existing works^26^. Specifically, the terminal reward is 24 if the patient survives, otherwise -24. The Q learning rate is 0.0001. We use the Python3.8 environment and PyTorch framework. All computations were performed on a PC equipped with a 3.30 GHz Intel Core i7-11370H CPU and 16 G RAM.

In model evaluation, the value of a newly learned AI policy is evaluated using trajectories of health status generated by another policy (the human clinicians). We employ an off-policy evaluation to evaluate the performance of each algorithm. In this paper, we use the double robust off-policy value evaluation^42^. The method calculates the unbiased estimator of strategies evaluated under each trajectory in Eq. (10) which combines importance sampling (IS) and approximate Markov decision model.

10 $$V_{t + 1} = \hat V\left( {S_t} \right) + \rho _t\left( {r + \gamma V_t - \hat Q_{\left( {S_t,a_t} \right)}} \right)$$

where *ρ* denotes the importance ratio of AI policy *π*_1_ and clinician policy *π*_0_: $\rho = \pi _1/\pi _0$. $\hat V\left( {S_t} \right)$ is evaluation value. $\hat Q_{(S_t,a_t)}$ is the expected return on the action *a* taken in the state *S*_*t*_. Jiang et al. confirmed the reward *r* and the importance ratio *ρ* are independent^42^. Hence the expected return *V* under unbiased estimation is obtained.

**Fig. 7:**
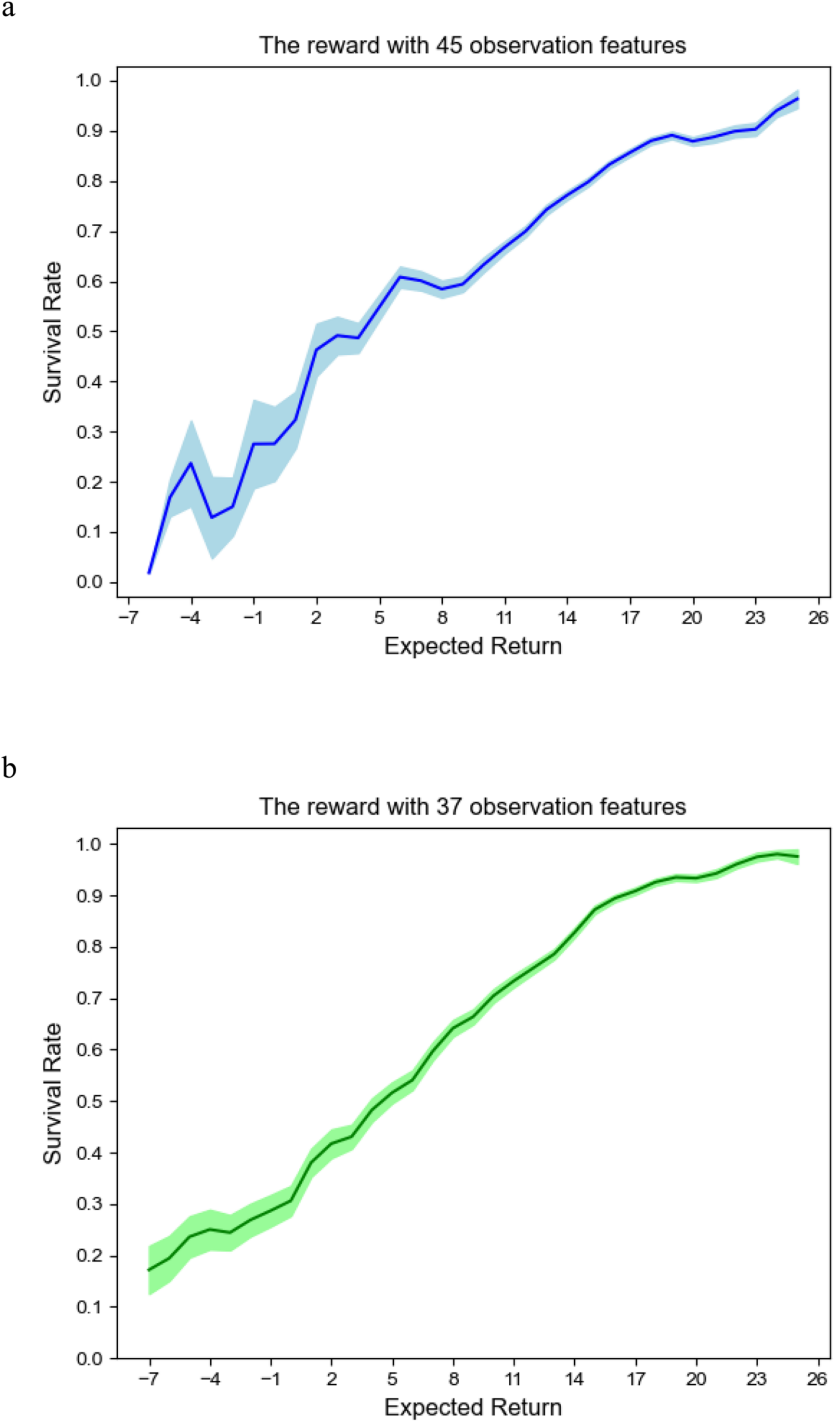
The relationship between expected return and survival rate. **a** The relationship between expected return and survival rate for 45 observation features. **b** The relationship between expected return and survival rate for 37 observation features. The relationship learned from observational data and actions taken by actual clinicians in the MIMIC-III dataset.

To further evaluate the policy survival rate, we apply an on-policy SARSA reinforcement learning algorithm ($Q\left( {S_t,a_t} \right) \leftarrow Q\left( {S_t,a_t} \right) + \alpha \left( {r + \gamma Q\left( {S_{t + 1},a_{t + 1}} \right) - Q\left( {S_t,a_t} \right)} \right)$ to establish the relationship between expected return and survival rate^29^. First, the expected return value *V* is calculated. Then, we calculate the average survival rate based on the return value. The survival formula^26^ Eq. (11) is shown below:

11 $$S\left( {Q_i} \right) = \frac{{sur_{V_i}}}{{tal_{V_i}}}$$

where $sur_{V_i}$ is the number of survivors and $tal_{V_i}$ is the total number of people given the expected return *V*_*i*_. *V*_*i*_ is an integer range of *V* and $V_t \in V_i$. The relationship between expected return and survival rate is shown in Fig. 7. The survival rate is positively correlated with the expected return for 45 and 37 observation features. We can see that the survival rate becomes greater as the expected return get increases.

### Reporting summary

Further information on research design is available in the Nature Research Reporting Summary linked to this article.

## Supplementary information

Supplementary Information

Reporting Summary

## Data Availability

The open-source MIMIC-III data used in this present study can be retrieved from https://physionet.org/content/mimiciii/1.4/.

## Code availability

The WD3QNE algorithm is implemented in the dueling deep Q network framework developed by the authors. Code and data are available at GitHub https://github.com/CaryLi666/ID3QNE-algorithm.
