# Supplementary material for: A value-based deep reinforcement learning model with human expertise in optimal treatment of sepsis
Source: NPJ Digit Med. 2023 Feb 2;6:15. doi: 10.1038/s41746-023-00755-5 (PMC9894526; doi:10.1038/s41746-023-00755-5)
Supplement: Supplementary file 1 — Supplementary Information [file 41746_2023_755_MOESM1_ESM.docx]

Supplementary Information

**Supplementary Note 1. Comparison of algorithm performance in OpenAI Gym LunarLander-v2 environments.**

In this paper, we develop a novel target Q value function method with adaptive dynamic weight (WD3QN). The method modifies the Q value function and is easily generalized to other value-based DRL methods. To verify the generality of the method, we use OpenAI Gym LunarLander-v2 environments^29^ to compare these methods. As shown in Supplementary Fig.1 (a), there is one space-ship as an agent. The task is to land the space-ship between the flags smoothly. There are four actions in it: do nothing, fire the left orientation engine, fire the downward engine, and fire the right orientation engine. The reward for moving from the top of the screen to the landing pad and coming to rest is about 100-140 points. If the ship crashes, it gets -100 points. If it comes to rest, it gets +100 points. The ship fires the downward engine getting -0.3 points or fires the side engine getting -0.03 points at each frame. At the end of each episode, the rewards for all steps in the episode are summed up to form an episode score. If the agent gets 200 points for 100 consecutive episodes, the experiment is forced to stop. The WD3QN method uses a smaller number of episodes (574) compared to D3QN (855), Dueling DQN (620), DDQN (720) and DQN (1000) in Supplementary Fig.1 (c). Supplementary Fig.1 (b) shows that Our method also consumes minimal time. Additionally, to further show the changes of adaptive dynamic weights $p$ value, we visualize the mean value and variance of $p$ variables in Supplementary Fig.2. we show that the mean value and variance of the $p$ varies through Q value weight updates for training episodes. We can clearly see that the mean of $p$ value goes down and then it goes up to 0.5. The variance of p value is relatively large at the beginning of training, and then gradually tends to 0. The novel structure of the target Q value function significantly improves the estimate accuracy of the target Q value and makes the algorithm converge faster.


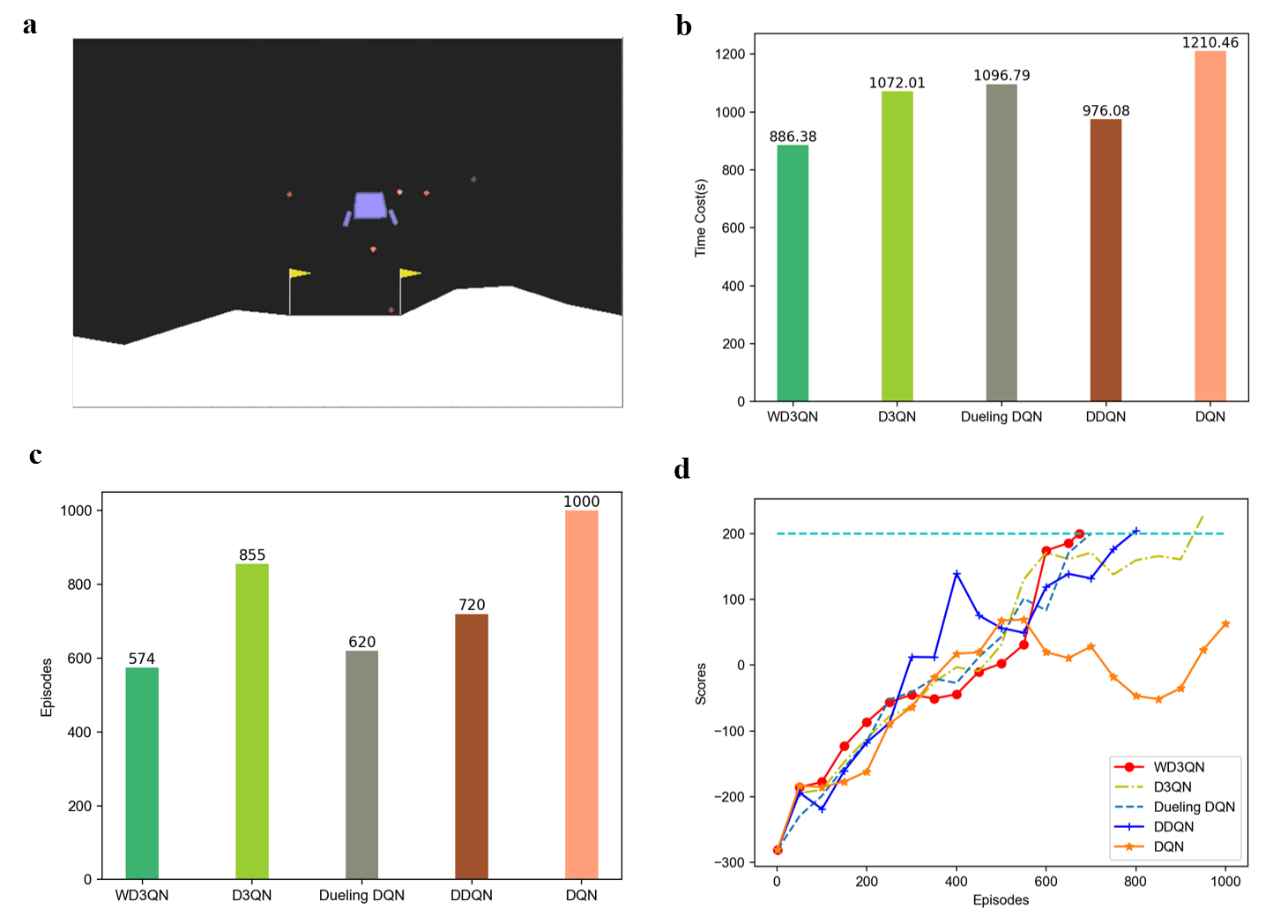


**Supplementary Fig. 1. Performance comparison of different methods. a** LunarLander-v2 environments. **b** Time cost of the algorithms. **c** Number of episodes reached +200 scores. **d** The training episode scores. The WD3QN method, which is target Q value function with adaptive dynamic weight into D3QN framework, is validated in the OpenAI Gym LunarLander-v2 environments. In all experiments, the parameters are the same and the experiment is forced to stop when the agent gets a reward of +200 for 100 consecutive episodes. We demonstrate that our method has better performance and generality.


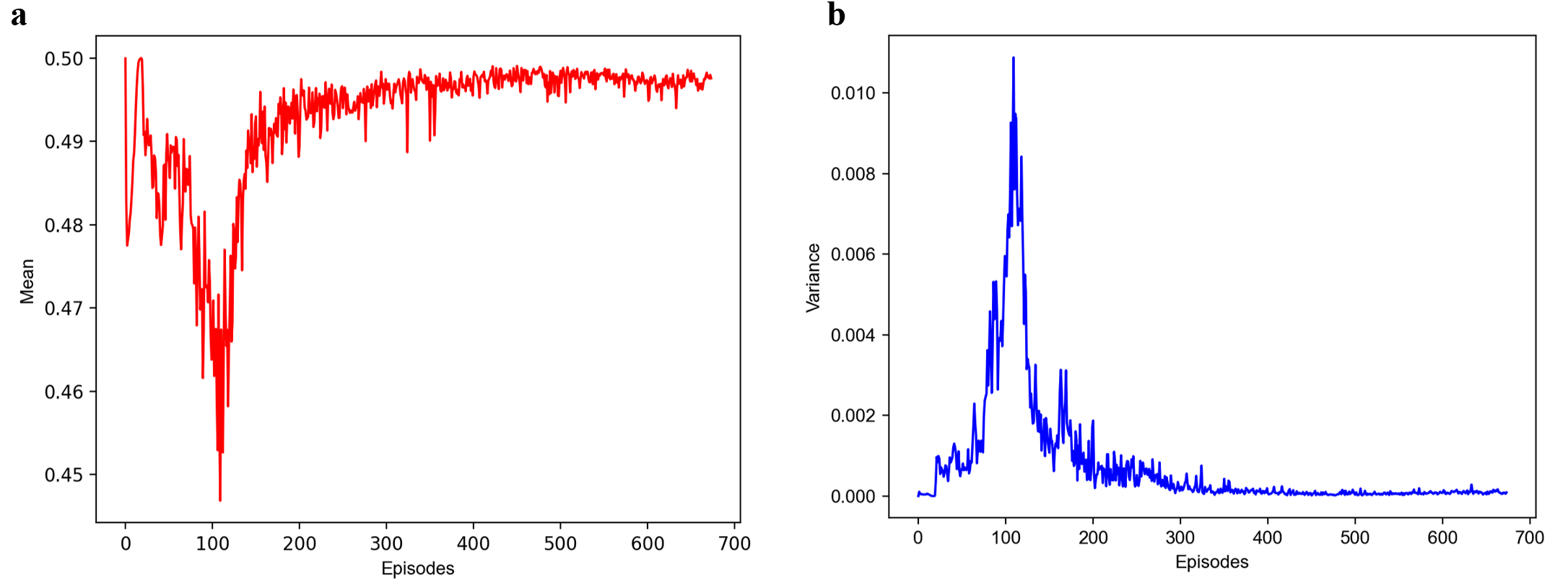


**Supplementary Fig. 2. Mean and variance of the**$\boldsymbol{p}$ **value.** a Mean of the $p$ value for episodes. **b** Variance of the $p$ value for episodes. The mean of the $p$ values is small at the beginning of training, which dynamically solves the overestimation of DQN methods. With the iteration training, $p$ value tends to 0.5. The adaptive dynamic weight $p$ effectively balances the overestimation and underestimation of DQN and DDQN methods. In addition, due to the early exploration of DRL algorithm, the variance of the $p$ value is relatively large and gradually tends to 0 with the training.

**Supplementary Tables.** Patient characteristics description of survivors and non-survivors.

**Supplementary Table 1.** **Demographics of the cohort for of survivors.**

| **Category** | **Feature** | **(Mean, SD)** | **Feature** | **(Mean, SD)** | **Feature** | **(Mean, SD)** |
| --- | --- | --- | --- | --- | --- | --- |
| Demographics | Survivors | 13,855 | Age | 63.1 (16.7) | Weight | 83.01 (24.7) |
|  | Male (N, %) | 7,675 (55.4%) | Race | [W, B, A, L, O] | |  |
| Vital signs | SOFA | 5.78 (3.13) | SIRS | 1.53 (1.03) | GCS | 12.87 (3.25) |
|  | HR | 86.45 (16.3) | SBP | 121 (20.09) | MBP | 79.07 (13.37) |
|  | DBP | 57.73 (13.25) | Shock Index | 0.73 (0.27) | SpO2(%) | 96.98 (2.38) |
|  | Temperature (℃) | 36.9 (1.13) |  |  |  |  |
| Lab values | Potassium | 4.06 (0.63) | Sodium | 138 (4.61) | Chloride | 104 (5.97) |
|  | Glucose | 6.33 (3.1) | BUN | 4.65 (20.76) | Creatinine | 0.71 (1.90) |
|  | Magnesium | 1.02 (0.53) | Calcium | 8.32 (0.82) | PaCO2 | 41.89 (10.4) |
|  | SGOT | 37.9 (12.3) | TB | 9.2 (4.61) | WBC | 7.9 (7.27) |
|  | Platelets | 233 (136.2) | PTT | 36.78 (18.32) | PT | 15.7 (6.11) |
|  | INR | 1.45 (0.70) | PH | 7.39 (0.02) | PaO2/FiO2 | 320 (229) |
|  | PaO2 | 98.2 (73.63) | HCO3 | 24.9 (4.85) | AL | 1.95 (1.49) |
|  | ArterialBE | 0.33 (5.0) | RR | 19.9 (5.01) | FiO2 | 0.45 (0.18) |
|  | SGPT | 31.5 (21.4) | HGB | 10.8 (0.72) |  |  |
| Fluid balance | Total input | 7960 (13008) | Total output | 6441 (12500) | 4Hourly output | 266 (522.8) |
|  | CB | 1513 (1284) |  |  |  |  |

**Supplementary Table 2.** **Demographics of the cohort for of non-survivors.**

| **Category** | **Feature** | **(Mean, SD)** | **Feature** | **(Mean, SD)** | **Feature** | **(Mean, SD)** |
| --- | --- | --- | --- | --- | --- | --- |
| Demographics | Non-survivors | 3,228 | Age | 70.1 (14.9) | Weight | 82.06 (25.5) |
|  | Male (N, %) | 1,853 (57.4%) | Race | [W, B, A, L, O] | |  |
| Vital signs | SOFA | 7.86 (3.98) | SIRS | 1.88 (1.03) | GCS | 11.69 (3.91) |
|  | HR | 89.39 (17.9) | SBP | 116 (20.73) | MBP | 75.7 (13.58) |
|  | DBP | 55.2 (13.5) | Shock Index | 0.79 (0.36) | SpO2(%) | 96.69 (3.44) |
|  | Temperature (℃) | 36.8 (3.56) |  |  |  |  |
| Lab values | Potassium | 4.12 (0.67) | Sodium | 139 (5.75) | Chloride | 104.7 (7.09) |
|  | Glucose | 5.53 (10.62) | BUN | 5.34 (26.51) | Creatinine | 0.83 (1.89) |
|  | Magnesium | 1.04 (0.53) | Calcium | 8.27 (0.91) | PaCO2 | 41.8 (11.7) |
|  | SGOT | 38.5 (12.8) | TB | 11.1 (6.4) | WBC | 10.2 (10.52) |
|  | Platelets | 214 (143.2) | PTT | 40.7 (21.93) | PT | 17.44 (7.93) |
|  | INR | 1.67 (0.96) | PH | 7.38 (0.36) | PaO2/FiO2 | 284 (202.9) |
|  | PaO2 | 119 (68.02) | HCO3 | 24.01 (5.66) | AL | 2.35 (2.19) |
|  | ArterialBE | -0.21 (5.46) | RR | 21.1 (5.60) | FiO2 | 0.48 (0.21) |
|  | SGPT | 32.1 (22.3) | HGB | 9.2 (0.73) |  |  |
| Fluid balance | Total input | 8308 (14238) | Total output | 6087 (13319) | 4Hourly output | 316 (639.1) |
|  | CB | 2220 (1470) |  |  |  |  |

Race: White, Black, Asian, Latino, Others; SOFA: Sequential Organ Failure Assessment; SIRS: Systemic Inflammatory Response Syndrome; GCS: Glasgow Coma Scale; HR: Heart Rate; SBP: Systolic Blood Pressure; MBP: Mean Blood Pressure; DBP: Diastolic Blood Pressure; BUN: Blood Urea Nitrogen; SGOT: Serum Glutamic-Oxaloacetic Transaminase; SGPT: Serum Glutamic Pyruvic Transaminase; TB: Total Bilirubin; WBC: White Blood Cells Count; PTT: Partial Thromboplastin Time; PT: Prothrombin Time; INR: International Normalized Ratio; PH: Arterial Potential of Hydrogen; PaO2/FiO2: PaO2/FiO2 Ratio; PaO2: Partial Pressure of O2; HCO3: Bicarbonate; AL: Arterial Lactate; ArterialBE: Arterial Base Excess; RR: Respiratory Rate; FiO2: Fraction of Inspiration O2; SGPT: Serum Glutamic Pyruvic Transaminase; HGB: Hemoglobin; CB: Cumulated Balance.
